# An integrative pipeline for circular RNA quantitative trait locus discovery with application in human T cells

**DOI:** 10.1093/bioinformatics/btad667

**Published:** 2023-10-31

**Authors:** Dat Thanh Nguyen

**Affiliations:** Centre for Integrative Genetics, Faculty of Biosciences, Norwegian University of Life Sciences, 1432 Ås, Norway

## Abstract

**Motivation:**

Molecular quantitative trait locus (QTL) mapping has proven to be a powerful approach for prioritizing genetic regulatory variants and causal genes identified by genome-wide association studies. Recently, this success has been extended to circular RNA (circRNA), a potential group of RNAs that can serve as markers for the diagnosis, prognosis, or therapeutic targets of various human diseases. However, a well-developed computational pipeline for circRNA QTL (circQTL) discovery is still lacking.

**Results:**

We introduce an integrative method for circQTL mapping and implement it as an automated pipeline based on Nextflow, named cscQTL. The proposed method has two main advantages. Firstly, cscQTL improves the specificity by systematically combining outputs of multiple circRNA calling algorithms to obtain highly confident circRNA annotations. Secondly, cscQTL improves the sensitivity by accurately quantifying circRNA expression with the help of pseudo references. Compared to the single method approach, cscQTL effectively identifies circQTLs with an increase of 20%–100% circQTLs detected and recovered all circQTLs that are highly supported by the single method approach. We apply cscQTL to a dataset of human T cells and discover genetic variants that control the expression of 55 circRNAs. By colocalization tests, we further identify circBACH2 and circYY1AP1 as potential candidates for immune disease regulation.

**Availability and implementation:**

cscQTL is freely available at: https://github.com/datngu/cscQTL and https://doi.org/10.5281/zenodo.7851982.

## 1 Introduction

Over the last two decades, genome-wide association studies (GWAS) have successfully detected thousands of DNA variants associated with human complex and disease traits (Visscher *et al.* 2017). Although the number of detected trait-associated variants continues to grow, understanding the underlying molecular mechanisms of most GWAS loci remains a challenge due to the non-coding nature of 90% GWAS loci (Hindorff *et al.* 2009, Tam *et al.* 2019). Among the possible approaches to tackle this challenge, molecular quantitative trait locus (QTL) mapping of genetic variants with intermediate molecular phenotypes, such as gene expression (eQTLs) and splicing (sQTLs), has proven to be a powerful tool to prioritize genetic regulatory variants and causal genes. Multiple studies have shown that GWAS hits are enriched in significant eQTLs/sQTLs loci and regulatory elements, suggesting gene regulation mechanisms of trait-associated DNA variants (Nicolae *et al.* 2010, Maurano *et al.* 2012, Walker *et al.* 2019, GTEx Consortium 2020, Kerimov *et al.* 2021, Mu *et al.* 2021). Moreover, the success of the QTL approach has recently expanded to include the discovery of genetic variants controlling RNA editing (Li *et al.* 2022).

Circular RNA (circRNA) is a relatively young class of RNA molecules characterized by covalently closed-loop structures without a 5′ cap or a 3′ poly (A) tail, formed by back-splicing events during RNA splicing processes (Jeck and Sharpless 2014, Chen *et al.* 2015, Meng *et al.* 2016). To date, over a million circRNAs have been identified in humans and other vertebrate species (Wu *et al.* 2020). Multiple studies showed that circRNAs exhibit unique expression patterns in tissues and developmental stages and are more stable than other RNA types (Salzman *et al.* 2012, 2013, Wang *et al.* 2014). Functional studies suggested that circRNAs play critical roles in various cellular processes and disease pathogenesis, including acting as microRNA sponges (Hansen *et al.* 2013, Memczak *et al.* 2013), regulating pre-mRNA splicing (Ashwal-Fluss *et al.* 2014), and modulating innate immunity (Liu *et al.* 2019b). Indeed, a few pioneer circRNA QTL (circQTL) studies shed the light on the impact of genetic variants on circRNA expressions to regulatory mechanisms underlying human complex diseases (Ahmed *et al.* 2019, Liu *et al.* 2019c, Mai *et al.* 2022, Aherrahrou *et al.* 2023).

While eQTL is well established with multiple computational pipelines and standardized protocols exist (Delaneau *et al.* 2017, GTEx Consortium 2020, Kerimov *et al.* 2021, Wang *et al.* 2021), circQTL mapping is still in its early stages with no comprehensive computational pipeline available. Current circQTL studies are commonly implemented by simply taking the output of a single circRNA detection algorithm, and directly using the BSJ counts as expression levels for QTL mapping (Ahmed *et al.* 2019, Liu *et al.* 2019c, Mai *et al.* 2022, Aherrahrou *et al.* 2023). This type of circQTL mapping is hereafter referred to as the single method approach. Although it is easy to use, utilizing only one circRNA calling method implies certain limitations. Firstly, circRNA detection still suffers from a certain amount of false positives regardless of the efforts of state-of-the-art circRNA calling methods (Zhang *et al.* 2016, Gao *et al.* 2018, Nguyen *et al.* 2021). Secondly, circRNA detection exhibits little agreement between calling tools that implies the potential divergence results in circQTL downstream analyses (Szabo and Salzman 2016, Hansen *et al.* 2015, Zeng *et al.* 2017, Hansen 2018).

To address these issues, we develop an integrative pipeline called cscQTL to systematical combine circRNA output from different tools for circQTL analysis by a re-quantification approach. Compared to the single method circQTL approach, cscQTL identifies more circQTLs and provides more coherence results. We implement cscQTL as an automated, reproducible, and scalable pipeline based on Nextflow (Di Tommaso *et al.* 2017). By applying cscQTL, we find genetic variants controlling expressions of 55 circRNAs in human T cells and identify circBACH2 and circYY1AP1 as potential circRNAs for immune disease regulation by colocalization tests.

## 2 Pipeline implementation

Motivated by previous studies demonstrating that combining multiple circRNA calling tools and re-quantification can improve consistency in circRNA calling and downstream differential expression analyses (Hansen 2018, Zhang *et al.* 2020), we propose an integrative pipeline called cscQTL (consensus-based circRNA QTL mapping) to address these challenges. Firstly, cscQTL improves the specificity by combining circRNA inputs from three high-accuracy circRNA identification algorithms. Secondly, cscQTL implements re-mapping and quantification procedures to provide accurate quantification of circRNAs. Finally, cscQTL is implemented as a non-stop pipeline (from circRNA detection, and genotyping data quality control to colocalization analysis) using Nextflow (Di Tommaso *et al.* 2017), which enables reproducible and scalable circQTL analyses in an automatic and user-friendly manner.

**Figure 1.**
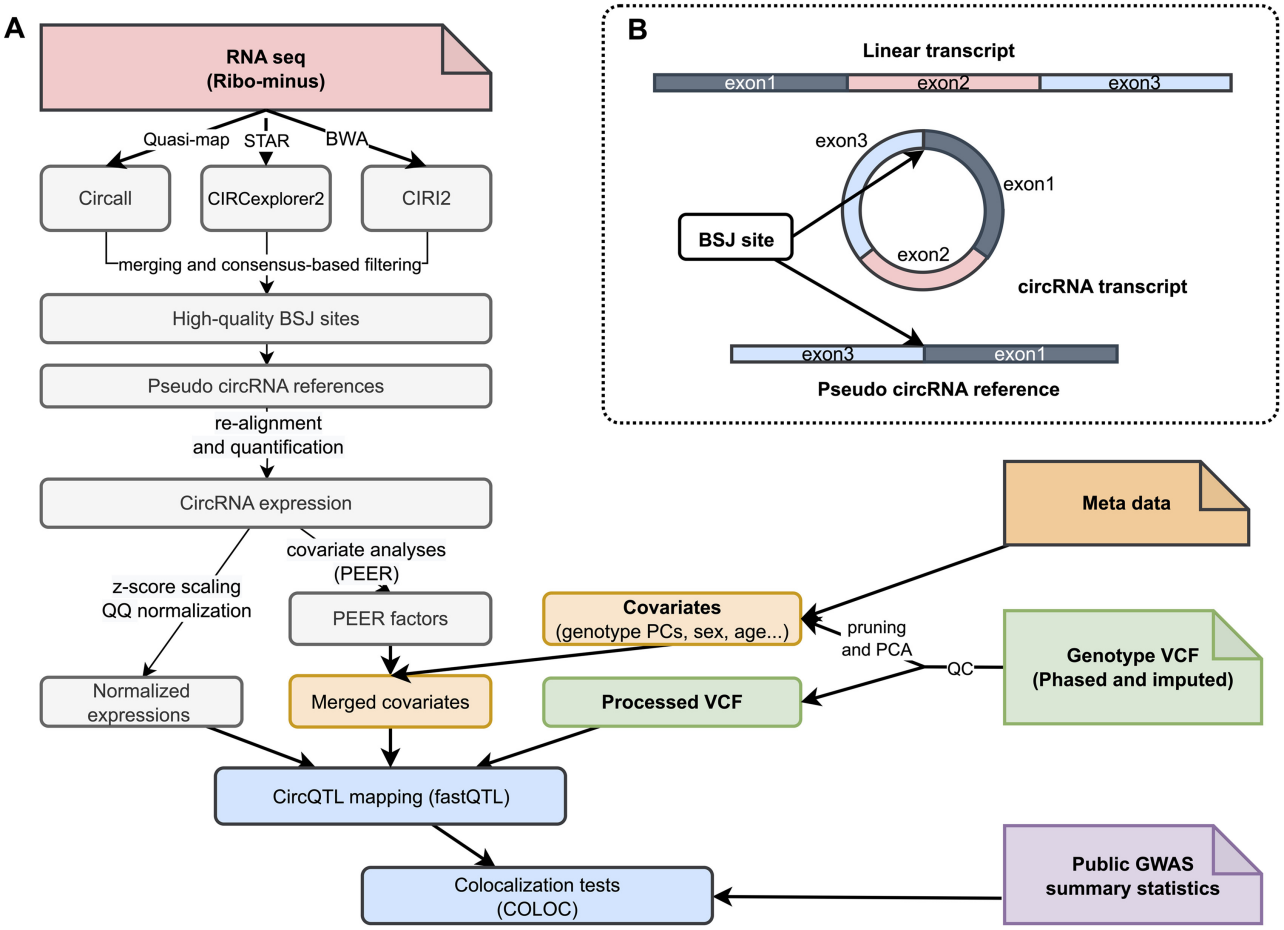
Overview of the cscQTL pipeline. (A) Workflow of circQTL mapping. CircRNAs are first identified by Circall, CIRCexplorer2, and CIRI2. Then, cscQTL applies consensus-based filtering to obtain high-quality circRNA candidates before quantifying by re-alignment of RNA-seq reads against the pseudo circRNA references; circRNA expressions then go through scaling, quantile–quantile normalization before CircQTL mapping and colocalization tests. (B) The construction of pseudo circRNA references. Based on circRNA annotations detected by circRNA calling algorithms, a pseudo circRNA reference is generated by joining 149 bases of the upstream sequences of the end positions with 149 bases of the downstream sequences of the start positions. It is noted the pseudo circRNA reference is not present in the corresponding linear form.

An overview of cscQTL is presented in Fig. 1A. First, RNA-seq (ribo-minus) data are used for circRNA identification by Circall, CIRI2, and CIRCexplorer2, with specific aligners allocated based on the authors’ suggested parameters. The detected circRNA candidates are harmonized to obtain a similar format before consensus-based filtering to obtain high-quality BSJ sites. In this implementation, circRNA candidates with ≥2 BSJ reads in at least one sample are kept before consensus filtering with different cutoffs of 1, 2, and 3 supporting methods. To accurately quantify the expression level of circRNAs and filter false-positive BSJ reads, reads that are fully mapped to the linear transcripts are discarded, and quantification is performed by counting the number of fragments mapped to pseudo circRNA references generated by concatenating 149 bases of the upstream sequences of the end positions with 149 bases of the downstream sequences of the start positions (Fig. 1B). Quasi-mapping is used for these alignment steps to ensure computational efficiency (Srivastava *et al.* 2016). Since only highly confident circRNAs are considered in the quantification, a loose filtering criterion is applied, i.e. filtering is applied with only one condition that the shorter piece of the read must cover the junction break point with at least seven bases to obtain the counting matrix. At the same time, input VCF genotype data, and metadata are also prepossessed. After that, the pipeline performs a series of processing steps including population filtering, z-score scaling, quantile–quantile (Q–Q) normalization, and covariate analyses with PEER (Stegle *et al.* 2012) before performing QTL mapping with FastQTL (Ongen *et al.* 2016) using the adaptive permutation scheme, and accounting for feature level multiple testing by the *q*-value procedure (Storey and Tibshirani 2003). Finally, the detected circQTLs are tested for colocalization with GWAS loci using COLOC (Hormozdiari *et al.* 2016). Further detailed descriptions are available in the Supplementary Material and method documents.

## 3 Application

To illustrate the efficiency of cscQTL, we apply the pipeline with different consensus cutoffs of 1, 2, and 3 denoted as cscQTL_1, cscQTL_2, and cscQTL_3, respectively, and compare them against the single method QTL approach that performs circQTL mapping by directly use circRNA quantification from a single circRNA calling method as implemented with Circall, CIRI2, and CIRCexplorer2, respectively. The comparison is performed using a publicly available dataset of 40 individuals with matched genotype and ribo-minus RNA-seq data (Chen *et al.* 2020) with uniform data processing procedures as described in detail in the Supplementary Material and method documents. Since we do not know the ground truth of genetic variants controlling circRNA expressions. We consider the result of the single method circQTL mapping approach as the baseline for evaluating the concordance and the number of eCircQTL called.

With the single method QTL approach, a total of 71 distinct eCircRNAs (circRNAs whose expression levels are associated with at least one genetic variant) are detected under Storey’s *q*-value <0.05 procedure (Storey and Tibshirani 2003). Among these, 46 were identified by Circall, 41 by CIRI2, and 15 by CIRCexplorer2. Less than 10% (7/71) of the detected eCircRNAs are supported by all three algorithms, and approximately one-third (24/71) is supported by at least two methods, as shown in Supplementary Fig. S1. These results indeed indicate limitations in both sensitivity and specificity of the single method circQTL mapping approach.

**Figure 2.**
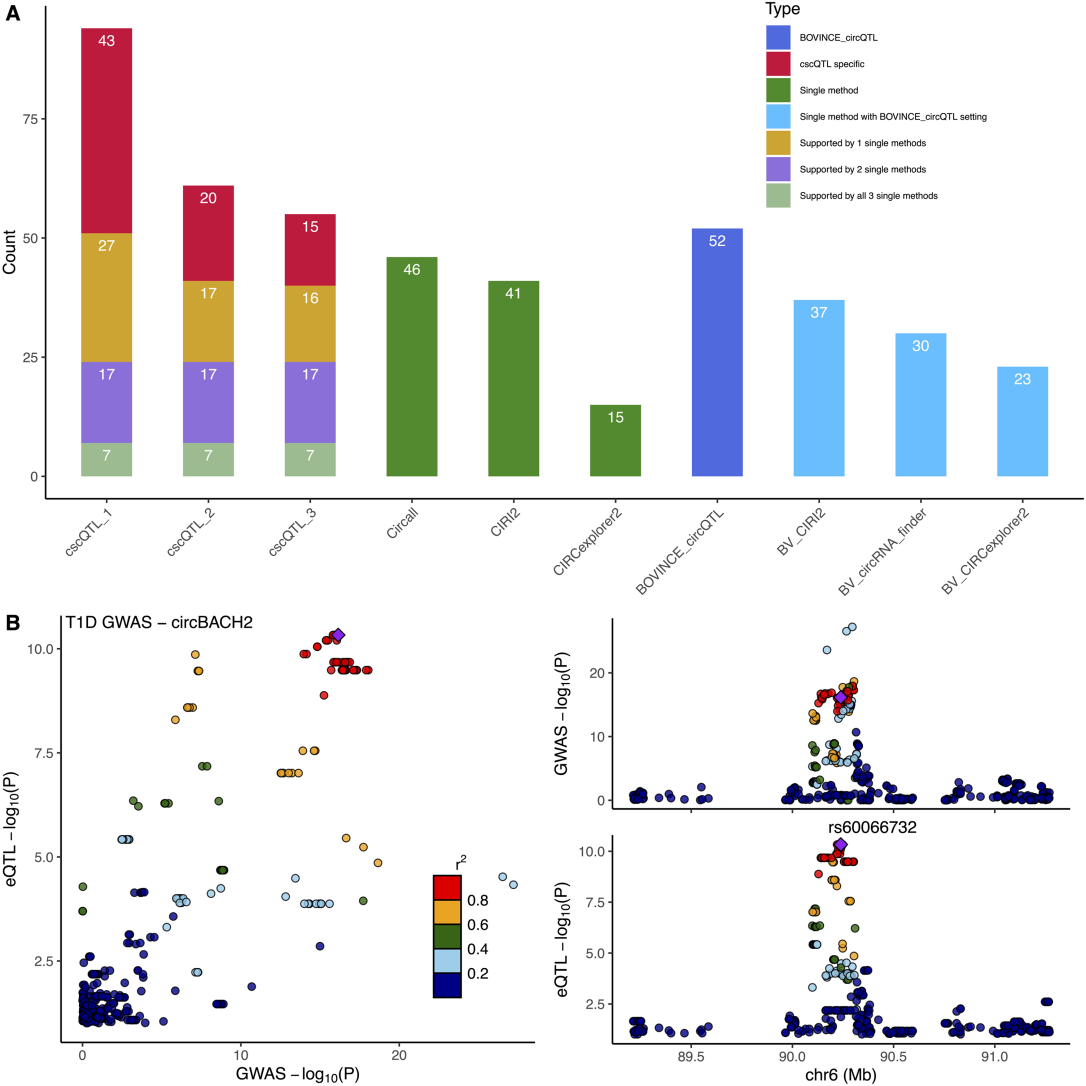
Result overview. (A). The number of eCircRNAs detected by Circall, CIRCexplorer2, CIRI, cscQTL (tested circRNAs are supported by at least 1, 2, and 3 circRNA detection algorithms corresponding to cscQTL_1, cscQTL_2, and cscQTL_3), BOVINE-circQTL and its components including BV_CIRI2, BV_CIRCexplorer, and BV_circRNA_finder. cscQTL results are shown as a breakdown of different eCircRNA classes that are supported by 3, 2, and 1 single methods and cscQTL-specific eCircRNAs. (B) Visualization of a T1D GWAS locus associated with circBACH2 (6:90206569:90271941).

Regarding cscQTL, the number of eCircRNAs detected is significantly higher than the single method approach as shown in Fig. 2A. At the loosest setting (consensus cutoffs of 1), cscQTL detected a total of 94 unique eCircRNAs. The corresponding numbers are 61, and 55 for the setting of 2, and 3 that cscQTL considers circRNA candidates supported by at least 2 or 3 methods by either Circall, CIRI2, and CIRCexplorer2 for re-quantification. Importantly, cscQTL results are highly concordant with the single method circQTL mapping. For instance, 40 out of 71 eCircQTL identified all three single methods are recalled by cscQTL_3. The corresponding number of cscQTL_2 and cscQTL_1 are 41 and 51 out of 71. Furthermore, all 24 highly confident eCircRNAs that are identified by at least 2 single methods are showing up in all consensus settings.

To further validate the new method, we conduct a comparison between cscQTL and another circQTL approach implemented in BOVINE-circQTL (available at https://github.com/luffy563/bovine_circQTL). Briefly, BOVINE-circQTL employs multiple tools for circRNA detection and conducts circRNA expression calibration using CIRIquant (Zhang *et al.* 2020) prior to QTL mapping for each circRNA tool. Detailed information regarding this comparison is provided in the supplementary documents. While BOVINE-circQTL successfully enhances the performance of individual methods, such as CIRCexplorer2, as demonstrated in Fig. 2A and Supplementary Fig. S2, it’s worth noting that the total number of eCircRNAs jointly identified by BOVINE-circQTL CIRI2 (BV_CIRI2), BOVINE-circQTL CIRCexplorer2 (BV_CIRCexplorer2), and BOVINE-circQTL circRNA_finder (BV_circRNA_finder) is still less than cscQTL with 52 compared to 55 eCircRNAs in cscQTL_3. Furthermore, we perform an extensive simulation (details available in the Supplementary Documents) to assess the quantification procedure of cscQTL (referred to as Circall_quant) in comparison to the state-of-the-art method CIRIquant (Zhang *et al.* 2020). The results demonstrate that both Circall_quant and CIRIquant achieve a high level of concordance with the ground truth number of circRNA transcripts, with Pearson correlation coefficients (*R*) of 0.9383 and 0.9184, respectively (see Supplementary Fig. S3A and B). Additionally, the concordance between Circall_quant and CIRIquant is remarkably high, with *R* = 0.9742, while the computational cost of Circall_quant is significantly lower than that of CIRIquant (see Supplementary Fig. S3C and D). These results collectively highlight the robustness of cscQTL.

To investigate the possible relation between circQTLs and immune GWAS loci, we further perform colocalization tests using COLOC (Hormozdiari *et al.* 2016) for eCircRNAs detected by cscQTL_3. Specifically, we obtain GWAS summary statistics of Crohn’s disease (CD), Inflammatory bowel disease (IBD) (Liu *et al.* 2015), and Type 1 diabetes (T1D) (Chiou *et al.* 2021) from the GWAS catalog webpage (MacArthur *et al.* 2017). We consider a PP.H4 ≥0.5 as the colocalization threshold and visualize the colocalization using LocusCompare (Liu *et al.* 2019a). Overall, 2 out of 55 circRNAs exhibit colocalization with immune disease GWAS loci including circBACH2 (6:90206569:90271941—ENSG00000112182) and circYY1AP1 (1:155676548:155679512—ENSG00000163374). circBACH2 is colocalized with all tested traits including T1D, CD, and IBD with probabilities of 0.89, 0.91, and 0.91, respectively (Fig. 2B, Supplementary Figs S4 and S5). Regarding circYY1AP1, it is colocalized to CD with a probability of 0.68 and to IBD with a probability of 0.61 (Supplementary Figs S6 and S7). Interestingly, BACH2 is a known risk gene for T1D (Marroquí *et al.* 2014) and circBACH2 in a well-known pathogenic circRNA (Cai *et al.* 2019). Overall, these results indicate the potential role of circRNAs in immune disease regulation.

## 4 Discussion

CircRNA detection is known as a challenging task. Deploying a single algorithm in a circQTL study indeed exhibits highly divergent results suggesting limitations in both sensitivity and specificity of the approach. Combining several algorithms in circRNA detection has been proposed and implemented in database construction (Hansen 2018, Wu *et al.* 2020) as an efficient solution for these issues. In this study, we extend this idea to circQTL analysis by developing a novel computational framework called cscQTL to unify outputs of multiple circRNA calling algorithms for circQLT mapping. By using a consensus-based filter together with the re-quantification procedure, cscQTL provides a coherent interpretation of circQTLs between circRNA calling algorithms while it is still able to improve the specificity by combining multiple circRNA calling algorithms. Compared to the single-method circQTL mapping approach, cscQTL recalls all highly confident circQTLs (identified by at least two single methods). The method further identifies more circQTLs than any single-method circQTL mapping even with the most stringent setting (considers only circRNA candidates identified in all three algorithms), indicating its reliability and robustness. Finally, we deploy cscQTL on a human T cells dataset as a showcase and discover genetic variants that control the expression of 55 circRNAs. By colocalization tests, we further identify circBACH2 and circYY1AP1 as potential candidates for immune disease regulation.

The current study also has some weaknesses. First, circRNA detection is restricted to three high-performance tools and absolute quantification. To address these issues, we develop a customized sub-pipeline implemented in “cscQTL_bed.nf” which can accept input circRNAs in bed file format. This modification makes cscQTL applicable to other circRNA detection tools and facilitates relative quantification studies, particularly those interested in exploring the ratios between circRNAs and their linear cognates. In addition, the application of the current study is limited to human T cells with a dataset of 40 samples. Nevertheless, future research endeavors could expand the scope by including larger datasets and conducting further investigations into ratio-based QTL analyses.

## 5 Conclusion

With the increasing attention on circRNAs, the bioinformatics community would benefit from a unified but open-source and portable circQTL workflow. By taking advantage of Nextflow, we implemented cscQTL as an easy-to-use pipeline and made it freely available to the public. With inputs being a directory of RNA-seq data, a genome reference, GWAS summary statistic data, and a metadata input file, cscQTL automatically performs circRNA identification, filtering, re-quantification, QTL mapping, and colocalization tests. As the potential role of circRNA in human health and disease is becoming more appreciated, we believe that our proposed framework will facilitate the discovery of circQTLs in the near future.

## Supplementary Material

btad667_Supplementary_Data

## Data Availability

Data supporting the findings and source codes to generate figures for this study are available at: https://github.com/datngu/cscQTL_paper.
