# Supplementary material for: An integrative pipeline for circular RNA quantitative trait locus discovery with application in human T cells
Source: Bioinformatics. 2023 Oct 31;39(11):btad667. doi: 10.1093/bioinformatics/btad667 (PMC10636286; doi:10.1093/bioinformatics/btad667)
Supplement: btad667_Supplementary_Data [file btad667_supplementary_data.zip › supp_methods.pdf]

Supplementary Documents

1 Supplementary material and methods

1.1 Datasets

A publicly available RNA-depleted RNA sequencing dataset consisting of 40 individuals, including CD4+ T naive cells, are obtained from Synapse (accessible at https://doi.org/10.7303/syn22250947). Briefly, purified and enriched CD4+ T cell samples were processed using the Illumina TruSeq Stranded Total RNA Kit with Ribo-Zero Gold, and subsequently, sequenced on the Illumina HiSeq 2000 platform, as previously described[1], to produce 100bp paired-end (PE) reads. Corresponding whole-genome sequencing genotypes of matched individuals are also downloaded from Figshare (https://doi.org/10.6084/m9.figshare.12646238.v5), as provided in the same publication[1].

1.2 Circular RNA identification

The downloaded ribo-minus RNA-seq reads are used for circRNA calling using three different tools: Circall v1.0.1[2], CIRI v2.0.6[3], and CIRCexplorer2[4]. The reference genome, transcriptome, and genome annotation of hg38 version 106 are obtained from the Ensembl website (http://ftp.ensembl.org/pub/release-106/). All tools are run with their default suggested parameters and designed alignment algorithms. For instance, Circall v1.0.1 is executed with the option "-td TRUE" and its built-in quasi-mapping[5]; CIRI2 is applied with BWA-MEM[6] parameter "–T 19"; and CIRCexplorer2 is deployed with the STAR aligner[7] with options "–chimSegmentMin 10 –chimOutType Junctions," as suggested in a benchmarking study[8].

1.3 Data processing and QTL mapping

To ensure a fair evaluation between circRNA calling tools used in the study, all detected circRNAs are uniformly processed. Initially, the detected circRNAs from each tool are merged. To filter out lowly expressed circRNAs, we apply a population-scale filter to keep only those with supporting BSJ counts greater than or equal to 2 and detected in at least 30% of the 40 samples. The BSJ counts are then normalized by library sizes to obtain circRNA count per million units (CPM).

The resulting expression matrices are then scaled and quantile-quantile normalized to fit a normal distribution as implemented in standard eQTL workflows[9,10].

Regarding the genotyping data, variants with Hardy-Weinberg Equilibrium (HWE) p-values below 10-6 and minor allele frequency below 5% are removed using PLINK v1.9[11]. Additionally, genetic principal components (PCs) are calculated after pruning using the options "–indep-pairwise 200 50 0.25" to exclude potential confounding effects due to population genetic structure.

QTL mappings are then performed using FastQTL v2.184[12]. For each test feature (circRNA/mRNA), the adaptive permutation mode is used with the setting with options "–permute 1000 10000" within 1MB window "–window 1e6". The obtained FastQTL beta distribution approximated empirical p-values are then used to calculate trait-level q values[13] to account for multiple testing. A false discovery rate (FDR) < 0.05 is then applied to identify eCircRNA .i.e. circRNA with at least one significant circQTL. For covariate inclusion in the linear regression, sex, age, four genotype PCs, and a number of PEER factors (between from 1 to 20)[14] are included to optimize the number of significant eCircRNA. The optimized PEER factor is then used for nominal QTL mapping to obtain nominal p-values for downstream analyses. Finally, the detected circQTLs are tested for colocalization with GWAS loci using COLOC[15]. In this implementation, we consider several immune disease GWAS summary statistics, including Crohn's disease, inflammatory bowel disease[16], and type 1 diabetes[17]. Significant GWAS loci are tested with all significant eCircRNAs with a threshold of PP.H4 $>= 0.5$.

1.4 BOVINE-circQTL comparison

To comprehensively validate cscQTL, we have undertaken a comparison with a recently developed circQTL pipeline known as BOVINE-circQTL (accessible at https://github.com/luffy563/bovine_circQTL). It is worth noting that this study has not yet been published, but the approach it employs is intriguing and warrants investigation. The primary concept behind the BOVINE-circQTL pipeline is to enhance circRNA expression quantification. This is achieved by first detecting circRNAs using various tools, including CIRI2[3], CIRCexplorer2[4], CircMarker[18], and circRNA_finder[19]. Subsequently, the expression levels are calibrated using CIRIquant[20] and then log-transformed before QTL mapping by MatrixEQTL[21].

Our focus is primarily on comparing strategies for circRNA detection and quantification, rather

than assessing different eQTL mapping methods. Therefore, we employ fastQTL[12], known for its effective eQTL mapping capabilities[22,23] for BOVINE-circQTL instead of MatrixEQTL as the original pipeline. In this context, BOVINE-circQTL utilizes three circRNA detection tools, namely CIRI2, CIRCexplorer2, and circRNA_finder (referred to as BV_CIRI2, BV_CIRCexplorer2, and BV_circRNA_finder, respectively). QTL mapping is carried out using fastQTL, applying the same multiple-testing correction procedure as cscQTL. These adjustments ensure a fair and equitable comparison between cscQTL and BOVINE-circQTL, as both pipelines utilize the same number of circRNA detection tools and employ a consistent QTL mapping procedure.

As a result, it seems that the calibration approach of BOVINE-circQTL effectively enhances the performance of single methods. In the case of CIRCexplorer2, as demonstrated in Fig 2 A and Fig S.2, both numbers of circRNAs after applying population filtering (BSJ >=2 in at least 30% sample size), and eCircRNA identified are sustainably increased. Regardless of this improvement, it's worth noting that the total number of eCircRNAs jointly identified by BV_CIRI2, BV_CIRCexplorer2), and BV_circRNA_finder is still less than cscQTL with 52 compared to 55 eCircRNAs in cscQTL_3.

1.5 Simulation studies

We compile a high-quality set of 14,904 circRNA candidates that are jointly detected by Circall, CIRI2, and CIRCexplorer2, using a back-splice junction (BSJ) cutoff of 2 in one of the 40 T cell samples for simulation purposes. To ensure the simulation closely resembled real data, we leverage Salmon[24] on a randomly selected sample (EGAR00001193090) to obtain expression values for linear transcripts. We then use the range from 0.2 to the 99th percentile of the observed expression distribution (excluding outliers) to randomly assign expression levels for the simulated dataset. Next, we employed Circall-simulator[2] and Polyester[25] to generate RNA-seq data for both circRNAs and the background linear RNAs. These simulations are configured with specific settings: a sequencing error rate of 0.005, a read length of 100 base pairs, a fragment length distribution with a mean of 250, and a standard deviation of 25. In total, we simulated 2,055,903 read pairs for circRNAs and 40,058,492 read pairs for linear transcripts. These reads are then combined to create the final simulated dataset containing both circRNAs and background linear transcripts.

To assess the performance of the customized quantification approach implemented in cscQTL, referred to as Circall_quant, we conduct a comparative analysis with CIRIquant v1.0.1 using default parameters. Both tools are executed with computational resources consisting of 32 cores of Xeon(R) CPU E5-2683 v4 and 64 GB of RAM. An input list of circRNA candidates is provided for both tools. To ensure a fair comparison, we zero-imputed the BSJ count outputs for circRNAs that were not initially detected by either tool. Subsequently, we apply a log(2+1) transformation to the BSJ count. We then compute Pearson correlation coefficients (R) to compare the estimated BSJ counts with the ground truth number of circRNA transcripts.

Overall, both Circall_quant and CIRIquant demonstrate a high degree of concordance with the ground truth number of circRNA transcripts, yielding R values of 0.9383 and 0.9184, respectively (refer to Fig S3 A and Fig S3 B). Notably, the concordance between Circall_quant and CIRIquant is remarkably high, achieving an R value of 0.9742 (Fig S3 A). Furthermore, it is important to highlight that the computational cost of Circall_quant is significantly lower than that of CIRIquant (see Fig S3 D).

References

[1] Chen, L. *et al.* Paired rrna-depleted and polya-selected rna sequencing data and supporting multi-omics data from human t cells. *Scientific Data* **7**, 376 (2020).

[2] Nguyen, D. T. *et al.* Circall: fast and accurate methodology for discovery of circular rnas from paired-end rna-sequencing data. *BMC bioinformatics* **22**, 1–18 (2021).

[3] Gao, Y., Zhang, J. & Zhao, F. Circular rna identification based on multiple seed matching. *Briefings in bioinformatics* **19**, 803–810 (2018).

[4] Zhang, X.-O. *et al.* Diverse alternative back-splicing and alternative splicing landscape of circular rnas. *Genome research* **26**, 1277–1287 (2016).

[5] Srivastava, A., Sarkar, H., Gupta, N. & Patro, R. Rapmap: a rapid, sensitive and accurate tool for mapping rna-seq reads to transcriptomes. *Bioinformatics* **32**, i192–i200 (2016).

[6] Li, H. Aligning sequence reads, clone sequences and assembly contigs with bwa-mem. *arXiv preprint arXiv:1303.3997* (2013).

[7] Dobin, A. *et al.* Star: ultrafast universal rna-seq aligner. *Bioinformatics* **29**, 15–21 (2013).

[8] Zeng, X., Lin, W., Guo, M. & Zou, Q. A comprehensive overview and evaluation of circular rna detection tools. *PLoS computational biology* **13**, e1005420 (2017).

[9] Li, Y. I. *et al.* Rna splicing is a primary link between genetic variation and disease. *Science* **352**, 600–604 (2016).

[10] Mu, Z. *et al.* The impact of cell type and context-dependent regulatory variants on human immune traits. *Genome biology* **22**, 1–28 (2021).

[11] Chang, C. C. *et al.* Second-generation plink: rising to the challenge of larger and richer datasets. *Gigascience* **4**, s13742–015 (2015).

[12] Ongen, H., Buil, A., Brown, A. A., Dermitzakis, E. T. & Delaneau, O. Fast and efficient qtl mapper for thousands of molecular phenotypes. *Bioinformatics* **32**, 1479–1485 (2016).

[13] Storey, J. D. & Tibshirani, R. Statistical significance for genomewide studies. *Proceedings of the National Academy of Sciences* **100**, 9440–9445 (2003).

[14] Stegle, O., Parts, L., Piipari, M., Winn, J. & Durbin, R. Using probabilistic estimation of expression residuals (peer) to obtain increased power and interpretability of gene expression analyses. *Nature protocols* **7**, 500–507 (2012).

[15] Hormozdiari, F. *et al.* Colocalization of gwas and eqtl signals detects target genes. *The American Journal of Human Genetics* **99**, 1245–1260 (2016).

[16] Liu, J. Z. *et al.* Association analyses identify 38 susceptibility loci for inflammatory bowel disease and highlight shared genetic risk across populations. *Nature genetics* **47**, 979–986 (2015).

[17] Chiou, J. *et al.* Interpreting type 1 diabetes risk with genetics and single-cell epigenomics. *Nature* **594**, 398–402 (2021).

[18] Li, X., Chu, C., Pei, J., Măndoiu, I. & Wu, Y. Circmarker: a fast and accurate algorithm for circular rna detection. *BMC genomics* **19**, 79–87 (2018).

[19] Westholm, J. O. *et al.* Genome-wide analysis of drosophila circular rnas reveals their structural and sequence properties and age-dependent neural accumulation. *Cell reports* **9**, 1966–1980 (2014).

[20] Zhang, J., Chen, S., Yang, J. & Zhao, F. Accurate quantification of circular rnas identifies extensive circular isoform switching events. *Nature communications* **11**, 90 (2020).

[21] Shabalin, A. A. Matrix eqtl: ultra fast eqtl analysis via large matrix operations. *Bioinformatics* **28**, 1353–1358 (2012).

[22] Consortium, G. The gtex consortium atlas of genetic regulatory effects across human tissues. *Science* **369**, 1318–1330 (2020).

[23] Kerimov, N. *et al.* A compendium of uniformly processed human gene expression and splicing quantitative trait loci. *Nature genetics* **53**, 1290–1299 (2021).

[24] Patro, R., Duggal, G., Love, M. I., Irizarry, R. A. & Kingsford, C. Salmon provides fast and bias-aware quantification of transcript expression. *Nature methods* **14**, 417–419 (2017).

[25] Frazee, A. C., Jaffe, A. E., Langmead, B. & Leek, J. T. Polyester: simulating rna-seq datasets with differential transcript expression. *Bioinformatics* **31**, 2778–2784 (2015).
