# Supplementary material for: An integrative pipeline for circular RNA quantitative trait locus discovery with application in human T cells
Source: Bioinformatics. 2023 Oct 31;39(11):btad667. doi: 10.1093/bioinformatics/btad667 (PMC10636286; doi:10.1093/bioinformatics/btad667)
Supplement: btad667_Supplementary_Data [file btad667_supplementary_data.zip › supp_figures.pdf]

Supplementary Figures

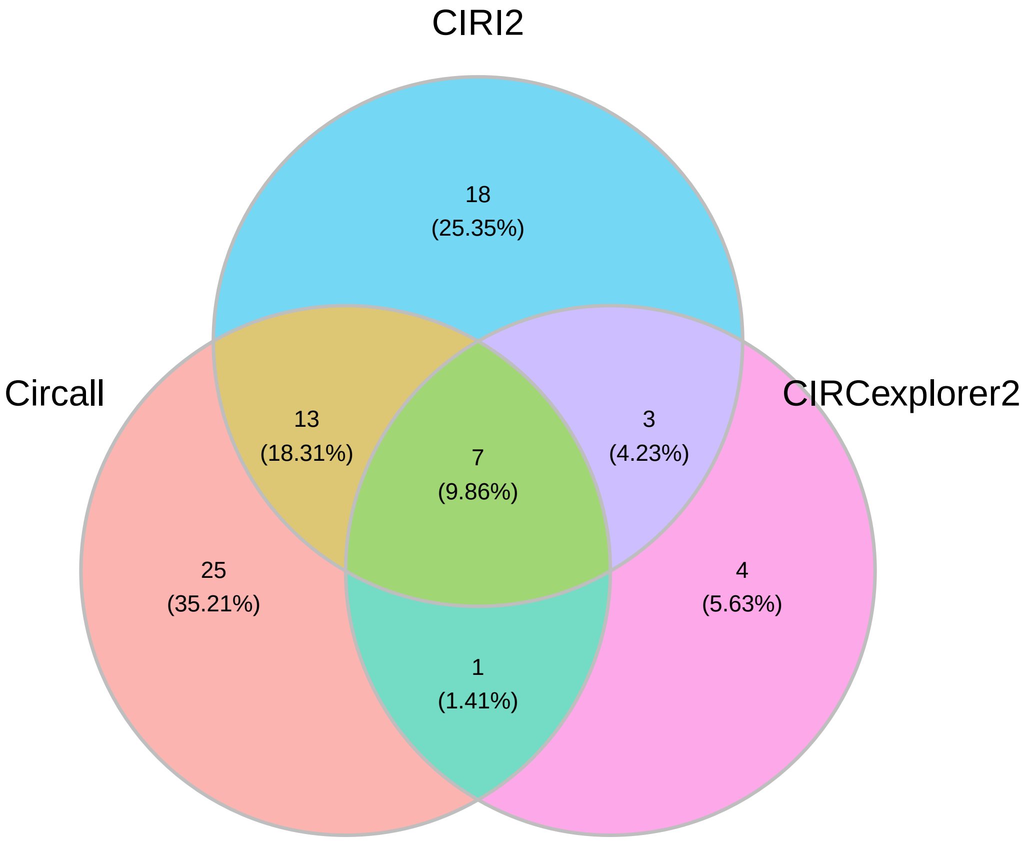

Fig S. 1 Venn diagrams showing the overlapping of eCircRNAs identified by the single method approach with three different circRNA calling algorithms.

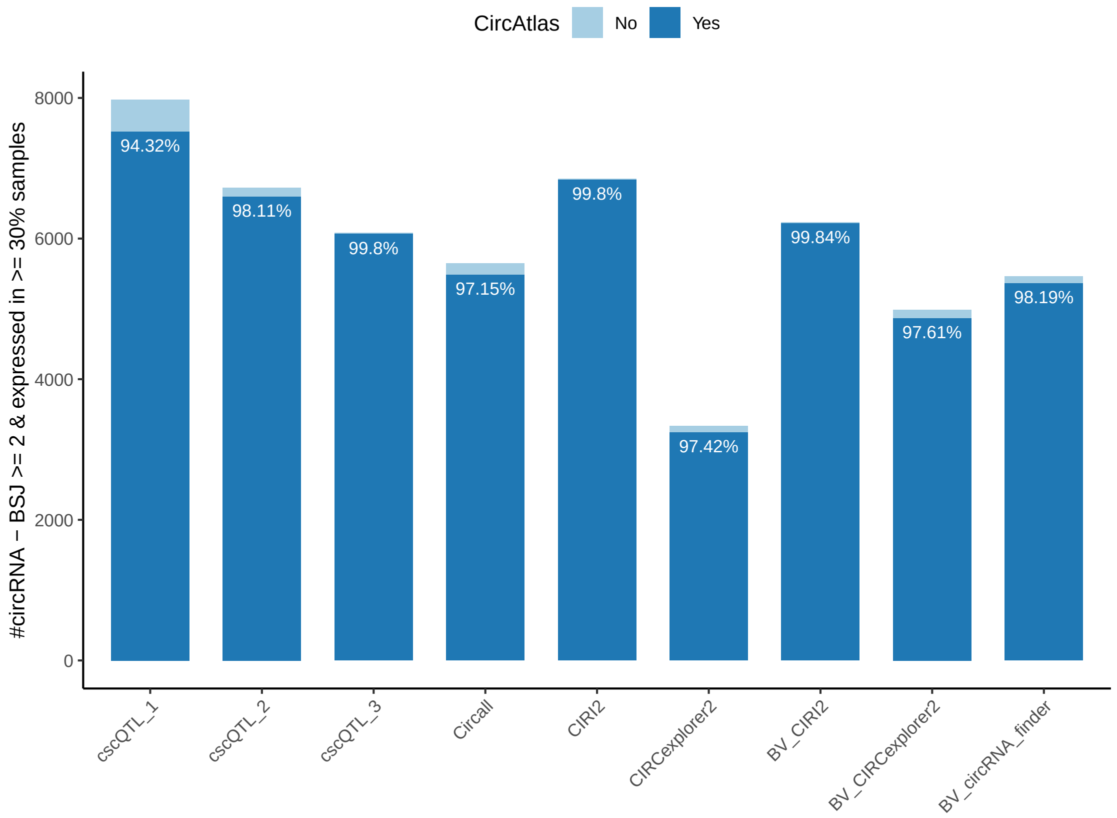

Fig S. 2 The number of circRNA after population filtering (BSJ counts >= 2 in at least 30% sample size) with reference to CircAtlas of cscQTL: consensus cutoffs: 1,2,3; single methods: Circall, CIRI2, CIRCexplorer2; and BOVINE-circQTL: BV_CIRI2, BV_CIRCexplorer, BV_circRNA_finder.

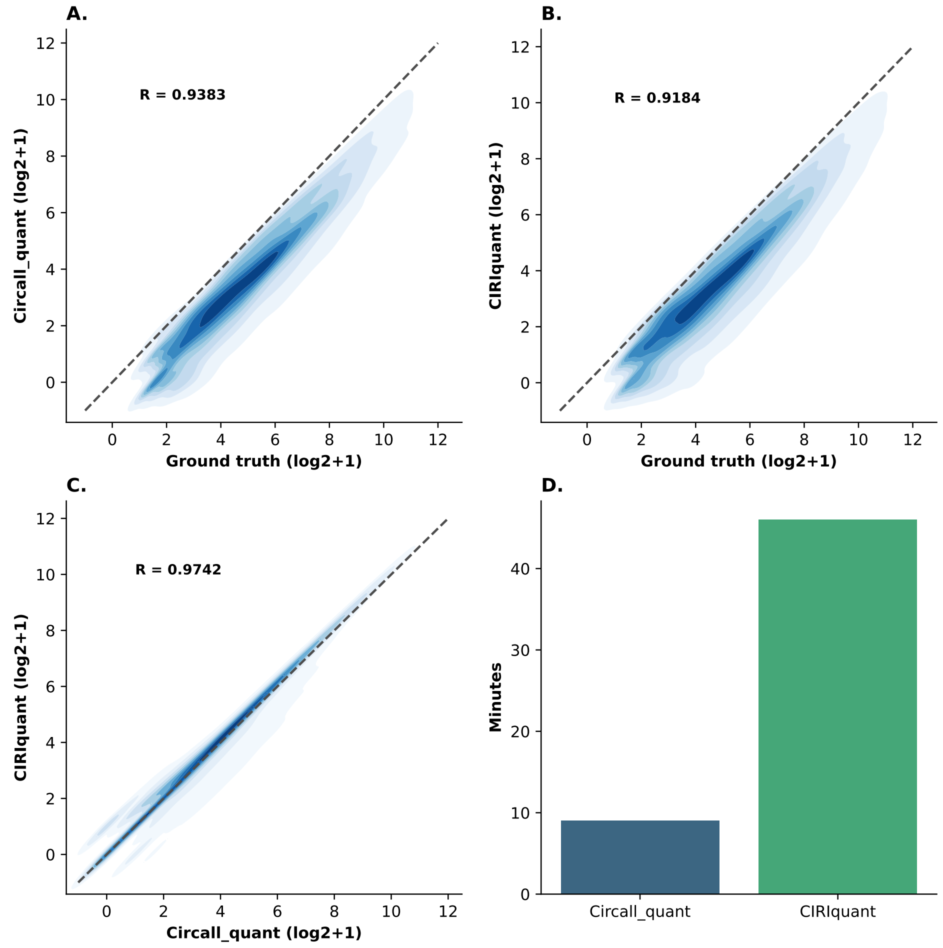

Fig S. 3 Simulation study results. (A), (B), (C) 2D density plot and Pearson correlation coefficient (log2 + 1 scale) of number simulated circRNA transcripts and BSJ counts detected by Circall_quant and CIRIquant. (D). Computational costs of Circall_quant and CIRIquant for the simulated dataset - with 32 CPUs and 64GB of RAM.

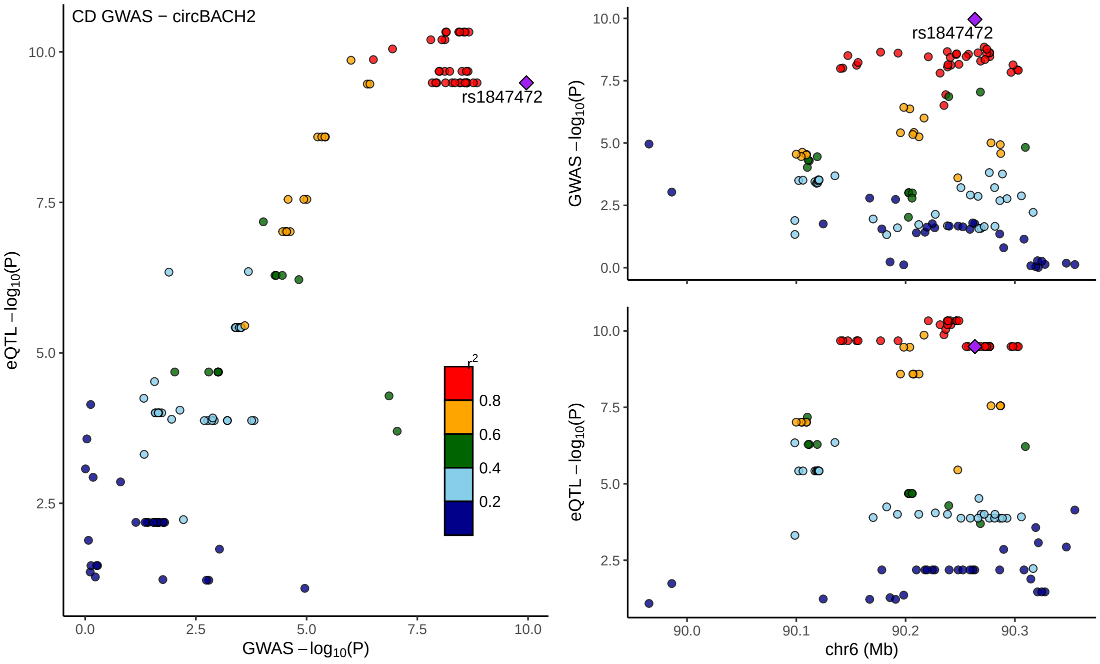

Fig S. 4 A CD GWAS locus associated with circBACH2 (6:90206569:90271941).

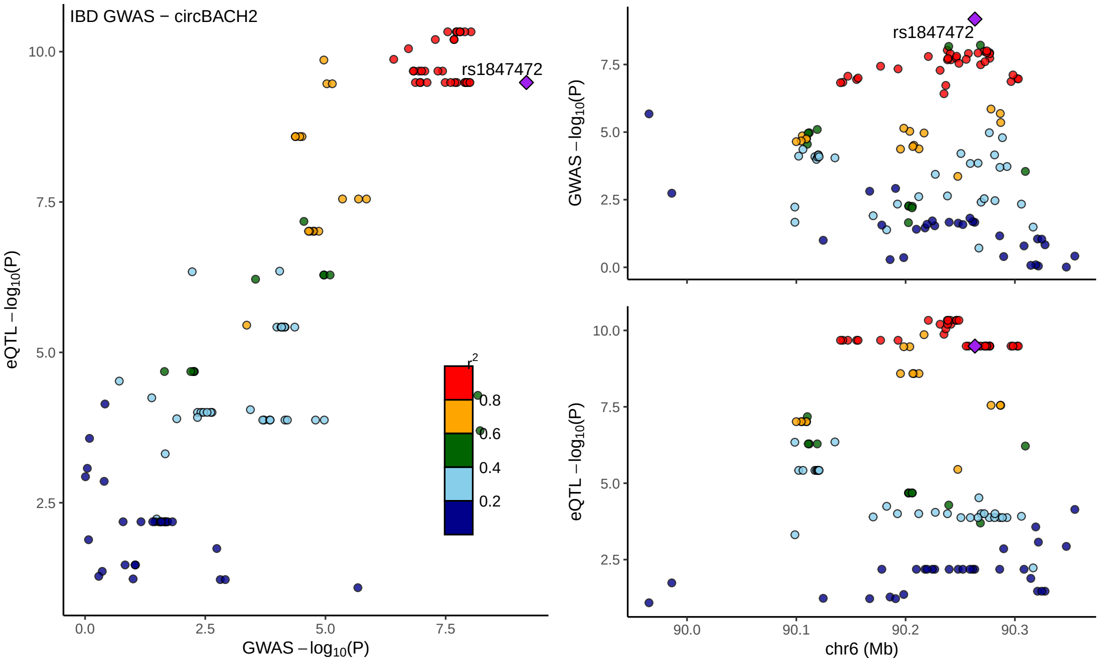

Fig S. 5 An IBD GWAS locus associated with circBACH2 (6:90206569:90271941).

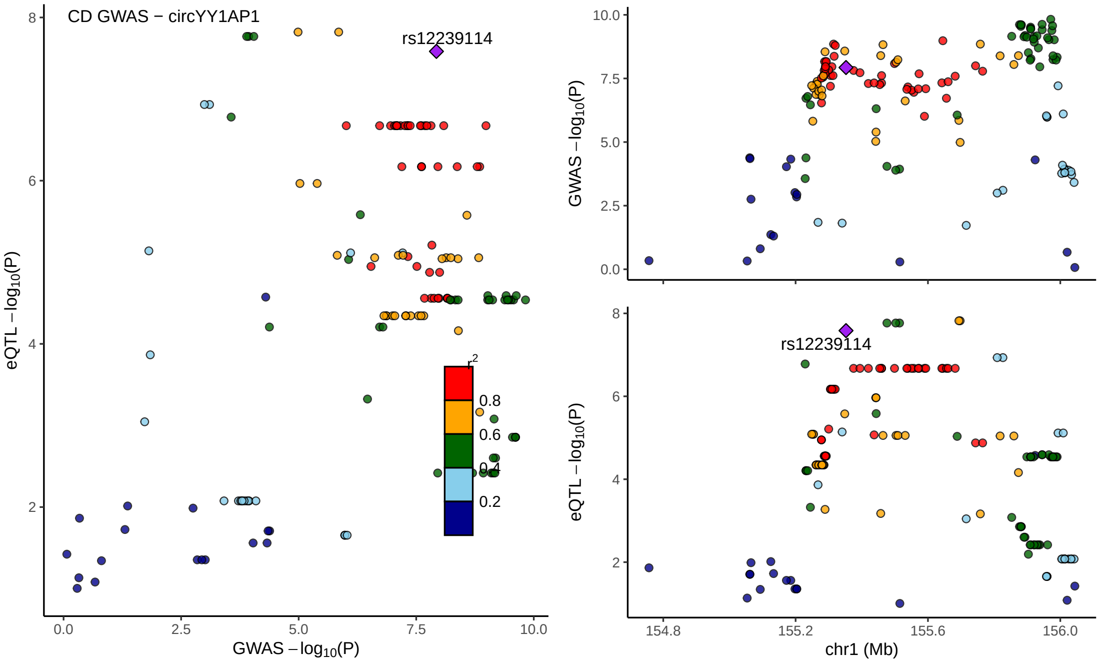

Fig S. 6 An CD GWAS locus associated with circYY1AP1 (1:155676548:155679512).

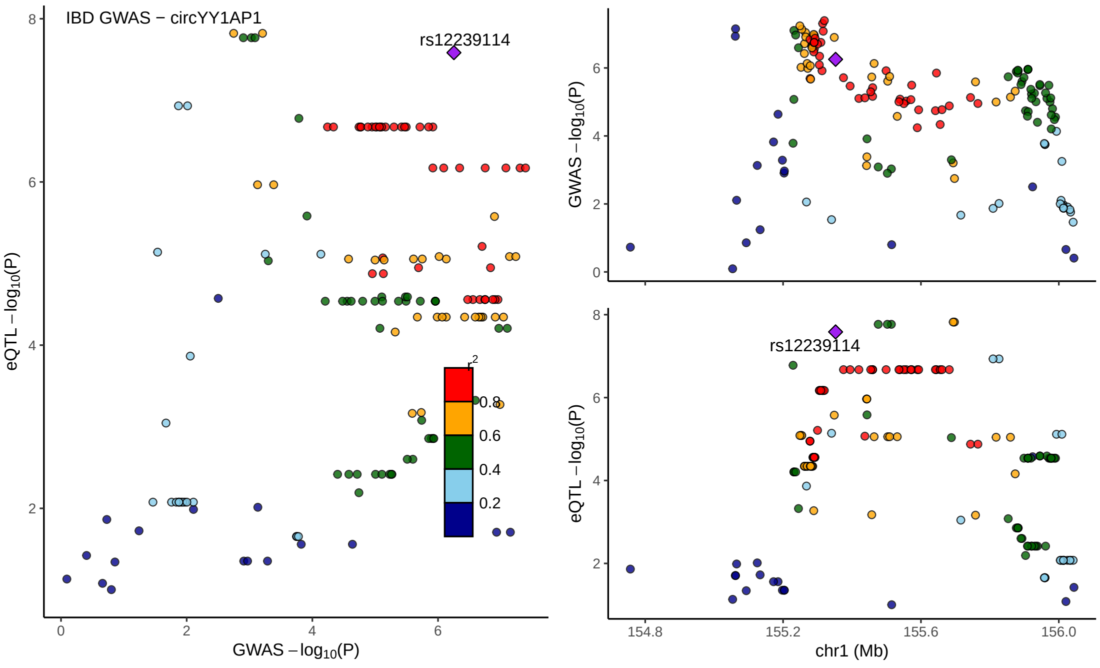

Fig S. 7 An IBD GWAS locus associated with circYY1AP1 (1:155676548:155679512).
